# The Reductive Dehydroxylation Catalyzed by IspH, a Source of Inspiration for the Development of Novel Anti-Infectives

**DOI:** 10.3390/molecules27030708

**Published:** 2022-01-21

**Authors:** Hannah Jobelius, Gabriella Ines Bianchino, Franck Borel, Philippe Chaignon, Myriam Seemann

**Affiliations:** 1 Equipe Chimie Biologique et Applications Thérapeutiques, Institut de Chimie de Strasbourg UMR 7177, Université de Strasbourg/CNRS, 4, rue Blaise Pascal, 67070 Strasbourg, France; hjobelius@unistra.fr (H.J.); gibianchino@unistra.fr (G.I.B.); p.chaignon@unistra.fr (P.C.); 2 Institut de Biologie Structurale, Université Grenoble Alpes/CEA/CNRS, 38000 Grenoble, France; franck.borel@ibs.fr

**Keywords:** MEP pathway, antibiotics, IspH, LytB, [4Fe-4S] cluster, reductive dehydroxylation, bioorganometallic intermediate, inhibitors

## Abstract

The non-mevalonate or also called MEP pathway is an essential route for the biosynthesis of isoprenoid precursors in most bacteria and in microorganisms belonging to the Apicomplexa phylum, such as the parasite responsible for malaria. The absence of this pathway in mammalians makes it an interesting target for the discovery of novel anti-infectives. As last enzyme of this pathway, IspH is an oxygen sensitive [4Fe-4S] metalloenzyme that catalyzes 2H^+^/2e^−^ reductions and a water elimination by involving non-conventional bioinorganic and bioorganometallic intermediates. After a detailed description of the discovery of the [4Fe-4S] cluster of IspH, this review focuses on the IspH mechanism discussing the results that have been obtained in the last decades using an approach combining chemistry, enzymology, crystallography, spectroscopies, and docking calculations. Considering the interesting druggability of this enzyme, a section about the inhibitors of IspH discovered up to now is reported as well. The presented results constitute a useful and rational help to inaugurate the design and development of new potential chemotherapeutics against pathogenic organisms.

## 1. IspH, an Enzyme Involved in the Biosynthesis of Isoprenoids

**Figure 1:**
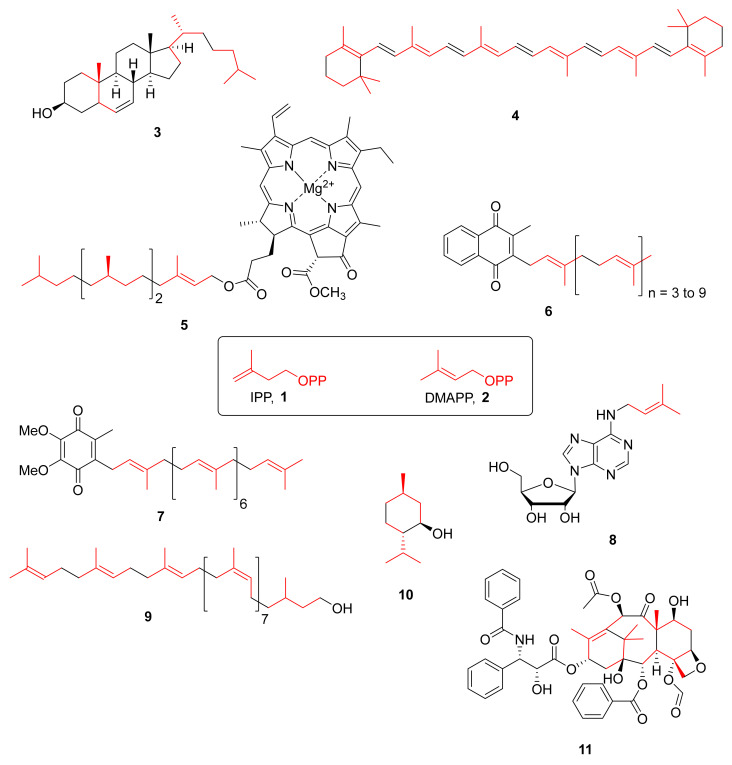
Some examples of isoprenoids and their biological precursors. The common five-carbon precursors are highlighted in red.

Isoprenoids, also known as terpenoids, constitute one of the largest classes of natural products with over 50,000 molecules. All terpenoids are derived from a common five-carbon precursor unit and are biosynthesized by the addition of one or more molecules of isopentenyl diphosphate (IPP, **1**) to its isomer dimethylallyl diphosphate (DMAPP, **2**) (Figure 1) [1]. The great structural diversity of isoprenoids is caused by different numbers of isoprene units, cyclization and oxidation reactions [2].

Isoprenoids are present in all living organisms and are involved in numerous important biological processes. For instance, in most eukaryotes, sterols as for example cholesterol **3** play the role of membrane stabilizers, and in vertebrates, they play the role of precursors of steroid hormones and bile acids. In phototrophic organisms, carotenoids (β-carotene, **4**) and chlorophylls (chlorophyll a, **5**) are essential for the conversion of light into chemical energy. Menaquinone **6** or ubiquinone **7** are used for electron transport in cellular respiration. Isopentenyladenosine **8** is a modification found in some tRNAs in bacteria and eukaryotes. Bactoprenol **9** is a crucial isoprenoid for bacteria as it is responsible for the transport of the biosynthetic sugar precursors of peptidoglycan, an essential constituent of the bacterial envelop.

From early on, humans took advantage of isoprenoids as odor compounds in fragrance or as essential oils with one famous representative being menthol **10**. More recently, taxol **11** was shown to be an efficient drug to treat some cancers.

Since the late 1950s, the mevalonate pathway was the only known metabolic pathway for the synthesis of IPP **1** and DMAPP **2** [3]. Importantly, this pathway is present in most eukaryotes including humans. One of its enzymes (HMGCoA reductase) is the target of drugs called statins that are given to patients suffering from hypercholesterolemia.

In the mid-1990s, an alternative pathway called the methylerythritol 4-phosphate (MEP) pathway was identified in bacteria, apicomplexan parasites and plant plastids for the biosynthesis of IPP and DMAPP [4,5,6].

**Scheme 1:**
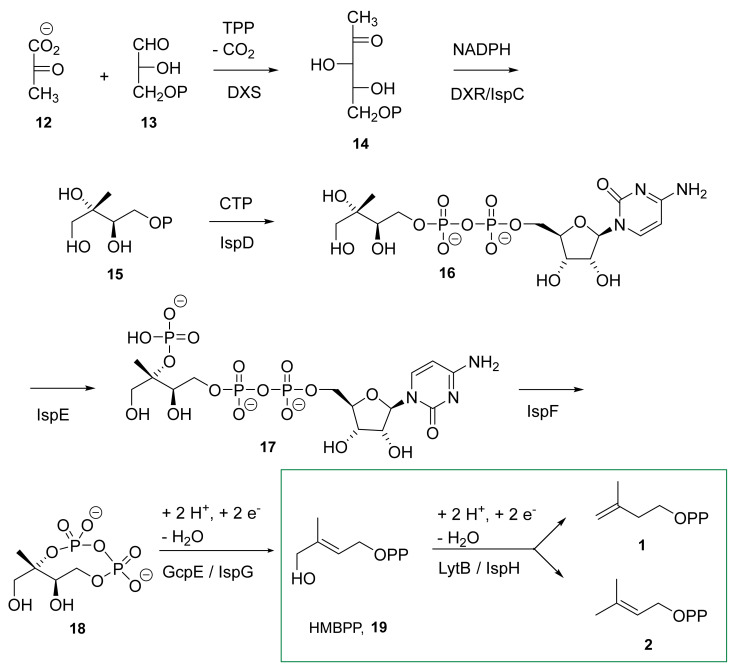
Methylerythritol phosphate pathway. The reaction catalyzed by IspH is framed.

The MEP pathway (Scheme 1) starts with the condensation of pyruvate **12** and glyceraldehyde 3-phosphate **13** to form 1-deoxy-D-xylulose-5-phosphate (DXP, **14**) catalyzed by the enzyme DXS. DXP is then converted into 2-*C*-methyl-D-erythritol-4-phosphate (MEP, **15**) by IspC (also called DXR). A sequence of three enzyme-catalyzed steps (IspD, IspE, and IspF) further converts MEP **15** into 2-*C*-methyl-D-erythritol-2,4-cyclodiphosphate (MEcPP, **18**) via cytidine diphosphate intermediates (4-diphosphocytidyl-2*C*-methyl-D-erythritol-2-phosphate **16** and 4-diphosphocytidyl-2*C*-methyl-D-erythritol-2-phosphate **17**). IspG/GcpE catalyzes the penultimate reaction by reducing and opening the cyclic diphosphate intermediate **18** to form (*E*)-4-hydroxy-3-methylbut-2-en-1-yl diphosphate (HMBPP, **19**).

In the last step of this biosynthetic route, IspH, also called LytB (EC.1.17.7.4), catalyzes the conversion of HMBPP **19** into a mixture of IPP **1** and DMAPP **2**. IspH, the subject of this review, catalyzes a two-electron reduction and OH-group elimination by using an oxygen sensitive [4Fe-4S]^2+^ center.

## 2. Occurrence of IspH

IspH as an enzyme of the MEP pathway occurs in most bacteria, plant chloroplasts, green algae, and apicomplexan but is absent in humans [7].

Interestingly, IspH is present in numerous disease-causing microbes including those posing a major challenge for new drug development. Indeed, antibiotic resistance is a growing concern as some infections have already become impossible to treat due to resistance. The worldwide emergence of carbapenemase-producing bacteria represents a health menace as carbapenems are often the last option for the treatment of patients infected by these bacteria [8]. Therefore, in 2017, the WHO published for the first time a list of antibiotic-resistant priority pathogens to guide research and development of new antibiotics as strains that cannot be fought by any antibiotic are emerging worldwide [8,9]. Importantly, 9 out of 12 of the classified bacteria contain an IspH enzyme (Table 1) highlighting its potential as a new target for antibiotic development.

*Mycobacterium tuberculosis*, the bacteria responsible for tuberculosis is another health threat. The WHO estimated that 1.8 million people worldwide died from tuberculosis each year with 250,000 cases from antibiotic resistant *M. tuberculosis* [8]. Interestingly, *M. tuberculosis* synthesizes its isoprenoids according to the MEP pathway and therefore relies on a functional IspH for survival. Actually, it was reported that *M. tuberculosis* contained two IspH homologs called LytB1 and LytB2 with both showing activity when expressed in *E. coli* [10]. However, it was shown that LytB2 was essential for *M. tuberculosis*’ viability as LytB1 was unable to complement for the loss of LytB2 [11].

Malaria is transmitted to humans through bites from female mosquitoes infected by the apicomplexan *Plasmodium falciparum* parasite and is causing nearly half a million deaths worldwide each year, mostly children less than 5 years of age [12]. In the context of fighting this life-threatening disease, fosmidomycin, an inhibitor of the *P. falciparum* enzyme DXR, the second enzyme of the MEP pathway, is under clinical trial as an antimalarial agent in combination with piperaquine [13], making the MEP pathway an attractive target for drug development. As an enzyme of the MEP pathway, IspH is not only present in *P. falciparum* but also in *Toxoplasma gondii*, the parasite responsible for toxoplasmosis.

Since the discovery of IspH two decades ago, an intense research activity has been devoted to the understanding of its mechanism with the aim of developing unprecedented therapeutics with new modes of action that are urgently needed to combat infectious diseases.

## 3. The Discovery of the IspH Metalloenzyme

### 3.1. The Lytb Gene

The *lytB* gene was first described in *Escherichia coli* by the group of Ishiguro in 1993 as a gene involved in penicillin resistance [15] and was further reported to be present in other bacteria [16]. The group of Gantt revealed that *lytB* was an essential gene for a *Synechocystis* strain (cyanobacterium) as its disruption was lethal and that the growth of this mutant could only be restored by the addition of the alcohol analogs of IPP and DMAPP (isopentenol and dimethylallyl alcohol) in the media. Furthermore, the overexpression of this *Synechocystis* gene in an *E. coli* mutant engineered to produce carotenoids led to increased accumulation of these isoprenoids in *E. coli* highlighting the role of *lytB* in isoprenoid biosynthesis and most probably in the MEP pathway [17]. *LytB* was further shown to be an essential gene for *E. coli* and to be involved in the MEP pathway [18,19]. The group of Jomaa reported that an *E. coli* mutant harboring a deletion of the *lytB* gene and engineered for the utilization of exogenous mevalonate (to rescue the cells and allow the synthesis of crucial isoprenoids when the endogenous MEP pathway is disrupted), accumulates an immunogenic compound stimulating the proliferation of Vγ9/Vδ2 T cells. This compound was identified as HMBPP **19** after its isolation followed by a complete characterization using NMR spectroscopy and mass spectrometry, affording the first evidence that HMBPP was the substrate of the *lytB* gene product [20].

### 3.2. The IspH Catalyzed Reaction

A very elegant in vivo approach was used by Rohdich, Eisenreich, and collaborators to identify the reaction catalyzed by the *lytB* gene product. An *E. coli* strain overexpressing the *xylB* gene coding for D-xylulose kinase, known to phosphorylate 1-deoxy-D-xylulose (DX) in DXP, and all the genes of the MEP pathway including *lytB* was constructed. After feeding of [U-^13^C_6_]DX to this strain followed by ^13^C-NMR analysis of the bacterial crude extract, it was shown that this *E. coli* mutant accumulated [U-^13^C_6_]IPP and [U-^13^C_6_]DMAPP in a 5:1 ratio [21]. When *lytB* was absent in the construction, the mutant accumulated [U-^13^C_6_]HMBPP from [U-^13^C_6_]DX [22]. Together, these results indicate that the *lytB* gene product, further called LytB or IspH, catalyzes the conversion of HMBPP into IPP and DMAPP in a mixture of 5:1.

*E. coli* IspH has a mass of about 36 kDa [21]. First activity assays performed with purified *E. coli* IspH displayed no activity whereas the cell-free extract of *E. coli* expressing the recombinant IspH was active suggesting the involvement of auxiliary enzymes [23]. Purified *E. coli* IspH was further shown to convert HMBPP into IPP and DMAPP with an activity of 3 nmol min^−1^ mg^−1^ when assayed under anaerobic conditions in the presence of a reduction system such as photoreduced deazaflavin or NADPH/flavodoxin/flavodoxin reductase [24]. An approximatively 3000 times higher activity was reported by Jomaa and collaborators for recombinant IspH from the thermophilic eubacteria *Aquifex aeolicus* when purified in a glove-box under anaerobic conditions and using reduced methyl viologen as reduction system [25]. The authors pointed out the fact that the enzyme’s activity could not be maintained after contact with oxygen indicative of the enzyme’s oxygen sensitivity.

### 3.3. IspH, a [4Fe-4S] Metalloenzyme

The nature of the iron-sulfur cluster of the IspH enzyme has been the subject of debate for about a decade. The cysteine residue is a common ligand for iron centers of which many examples are known in literature [26]. Rohdich et al. assumed the presence of such a cluster due to three conserved cysteines which they found in ten different species containing the IspH protein [23]. The iron-sulfur cluster of IspH was first experimentally evidenced in the UV visible spectrum as a broad absorption maximum at a wavelength of 420 nm [25]. By comparison with spectroscopic data of other holoenzymes such as IspG [27], which is the enzyme prior to IspH in the MEP pathway, a [4Fe-4S]^2^^+^ cluster was proposed [24].

The early propositions concerning the [4Fe-4S]^2+^ cluster were backed up by Rohmer and collaborators conducting EPR spectroscopic measurements after reconstitution of the Fe/S cluster by incubation of the apoenzyme with FeCl_3_, Na_2_S, and dithiothreitol under anaerobic conditions [28]. [4Fe-4S]^2+^ itself does not show any signals on the EPR spectrum due to its diamagnetic nature. Upon reduction of the chemically reconstituted enzyme with dithionite, however, a paramagnetic species appeared having *g* values of 1.92(1) and 2.03(7). By analogy to earlier studies of iron-sulfur clusters measured by EPR spectroscopy, the microwave power half-saturation P_1/2_ was used as means to distinguish between a [2Fe-2S]^+^ and a [4Fe-4S]^+^ species [29,30]. With a rather high P_1/2_ value of 153 mW, a [4Fe-4S]^+^ cluster was proposed [28]. Iron species that could be detected on the enzyme purified under aerobic conditions were attributed to [3Fe-4S]^+^ (*g* = 2.02) and to ferric ions (*g* = 4.29) and exhibited signals already known from literature [31]. The discussion about the nature of the iron-sulfur cluster continued owing to new results from Rohdich et al. [32]. In their work, recombinant *E. coli* IspH protein was expressed in the presence of an *isc* operon, enabling the assembly of the full iron-sulfur cluster in vivo, and purified under anaerobic conditions. They found the same absorption maximum in the UV visible spectrum at about 410 nm as had been found before [28], but with a lower absorption coefficient (here: *ε* = 11,800 M^−1^ cm^−1^, compared to *ε* = 18,750 M^−1^ cm^−1^ in the previous study) suggesting a lower concentration of the iron-sulfur cluster. The corresponding EPR spectrum showed an anisotropic signal with *g*-values at 2.032(3) and 2.003(3). Its symmetry led them to the conclusion that a [3Fe-4S]^+^ was the active species as they found a high catalytic rate of 550 nmol mg^−1^ min^−1^ when using NADPH/flavodoxin reductase/flavodoxin as reduction system.

In order to solve this controversy, two groups independently investigated *E. coli* IspH purified under anaerobic conditions using Mössbauer spectroscopy [33,34]. Irrespective of its magnetic state, Mössbauer spectroscopy offers the possibility to gain structural information about the iron-sulfur cluster [35]. The analyses of the Mössbauer spectra indicated concordantly the structure of a [4Fe-4S]^2+^ cluster with a unique fourth iron site. The spectra could be simulated by the sum of three components with a ratio of 2:1:1 as shown in Table 2 and indicated an unusual [4Fe-4S]^2+^ center [33]. Two iron centers share one electron with an oxidation state of +2.5 each as derived from their isomer shift of *δ* = 0.42 mm s^−1^ and exhibit a tetrahedral coordination sphere with four sulfur ligands; besides the three sulfide ions, a cysteine residue is coordinated to each site as confirmed by X-ray structure analysis [36,37]. The two remaining iron sites consist of a high-spin Fe^3+^ ion and a high-spin Fe^2+^ ion, respectively [33]. The latter was assumed to have a coordination sphere with five or six ligands, most likely three sulfur and three nitrogen or oxygen ligands. Schünemann and collaborators further used Mössbauer spectroscopy to unequivocally access the nature of the Fe/S cluster of IspH existing in vivo in *E. coli* [33]. The authors recorded Mössbauer spectra of *E. coli* cells overexpressing IspH and of a WT strain, both strains grown under the same conditions. The difference of the spectra obtained for each type of cells could be simulated using the Mössbauer parameters obtained for pure ^57^Fe IspH. This proves that IspH assembles a [4Fe-4S]^2+^ cluster in vivo that is maintained when the protein is purified under anaerobic conditions. More details about the coordination sphere on this unique fourth iron atom were revealed by nuclear resonance vibrational spectroscopy (NRVS) on substrate-free IspH by the groups of Schünemann and Seemann [38]. NRVS is an advanced Mössbauer technique using synchrotron based radiation that is sensitive to vibrational modes involving iron [39]. By comparison of the experimental spectrum with DFT calculations obtained on an IspH structure harboring an incomplete Fe/S cluster (PDB: 3F7T) in which they inserted the missing iron, the authors concluded that the unique iron site was in a hexa-coordinated form with three water molecules. Together, these results highlight that IspH harbors an unusual [4Fe-4S] cluster linked to the three conserved cysteines with a unique iron site of oxidation state +2 that is linked to three sulfides of the cluster and to three water molecules.

The fact that the [4Fe-4S] cluster was the active species in the IspH enzyme was further confirmed by Duin et al. [40]. They could show that the enzyme’s activity was linearly dependent on the content of its cofactor.

It is now firmly established that IspH is an oxygen sensitive [4Fe-4S]^2+^ enzyme that catalyzes a reductive dehydroxylation and that the electrons needed for this reduction are provided in vivo by a biological system such as NADPH/flavodoxin reductase/flavodoxin in the case of *E. coli* or NADPH/ferredoxin reductase/ferredoxin in the case of *P. falciparum* [41].

## 4. The Mechanism of the Reductive Dehydroxylation

### 4.1. Protein Conformation in the Crystalline State

**Figure 2:**
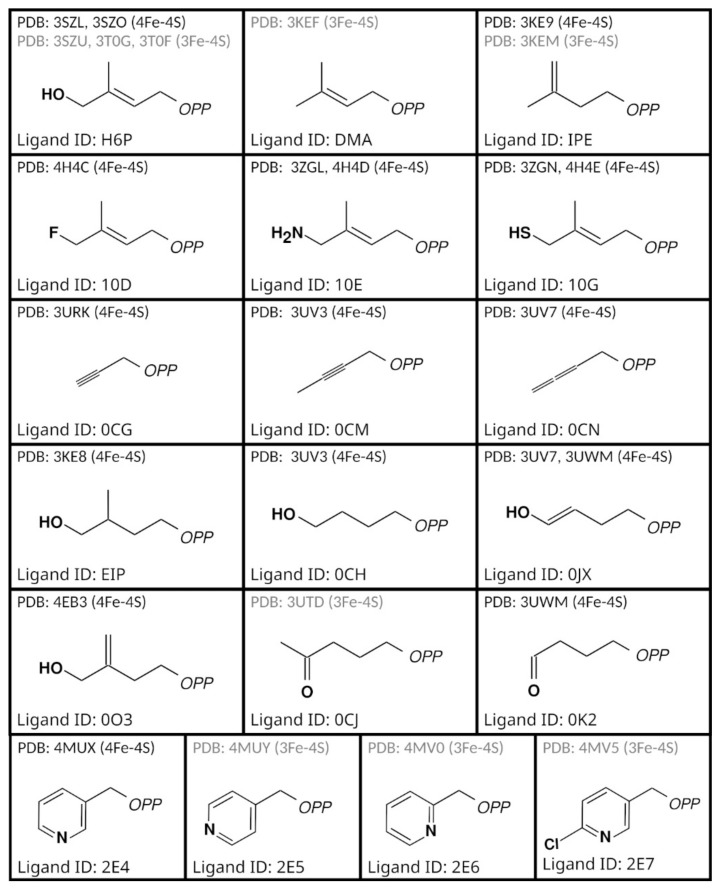
Structures of IspH deposited in the PDB database with their corresponding ligand.

Crystal structures can give hints for the elucidation of the mechanism, but the propositions are usually confirmed by studies in solution. During the last two decades, 30 IspH crystallographic structures were deposited into the PDB database (Figure 2, Table 3). IspH structures from three different organisms are available. The first structure solved at the end of 2008 by Ermler and collaborators was the one from *Aquifex aeolicus*, a Gram-negative hyperthermophilic bacteria [36]. In 2009, Gräwert et al. released the first IspH structure of *E. coli* [42]. The last organism for which the crystallographic structure of IspH has been described is the one from the apicomplexan parasite *P. falciparum* [43]. For *A. aeolicus* and *E. coli* the full-length protein was crystallized whereas for *P. falciparum* the 217 first amino acids were not included in the construct used for the production and the crystallization of the enzyme.

The unprecedented fold of all IspH enzymes looks like a three-leaf clover and is composed of three structurally similar domains that do not display detectable similarity [42]. Each domain comprises a central parallel beta sheet containing four beta strands surrounded by three or four alpha helices. The active site is buried in a hydrophobic cavity embedded between the three domains. The [4Fe-4S] cluster, required for enzyme activity [28], is bound to the protein via three highly conserved cysteines [42]. Each domain contributes to the cluster coordination since these cysteines are located at the N-terminus of the first alpha helix of each of the three domains. *E. coli* and *P. falciparum* display an additional extended C-terminal loop containing a short beta sheet positioned above the active site pocket to protect the cluster from the surrounding solvent. In *A. aeolicus*, the dimerization of the protein is proposed to achieve the same role [44]. However, it should be noted that the crystal structure of the thermophilic enzyme does not reveal evidence of dimerization [36].

Among the 30 IspH structures solved only the one of *A. aeolicus* was obtained without a ligand into the binding pocket. This structure contained an incomplete [3Fe-4S] cluster. It should be mentioned that no crystal structure of substrate-free IspH in its [4Fe-4S] form has been reported to date. The inability to obtain such a structure is most probably due to the instability of the apical iron linked to three water molecules [38], which dissociates during the crystallization process. Comparison of this empty structure with the others reveals a conformational change. Indeed, upon substrate binding, a motion of the third domain of IspH results in the closing of the active site [42], and from the *P. falciparum* structure [43] it appears that a single sulfate ion bound in the substrate cavity is sufficient to induce the closing of the active site.

### 4.2. First Step: Binding of the Hydroxyl Group of HMBPP to the Iron-Sulfur Cluster

**Scheme 2:**
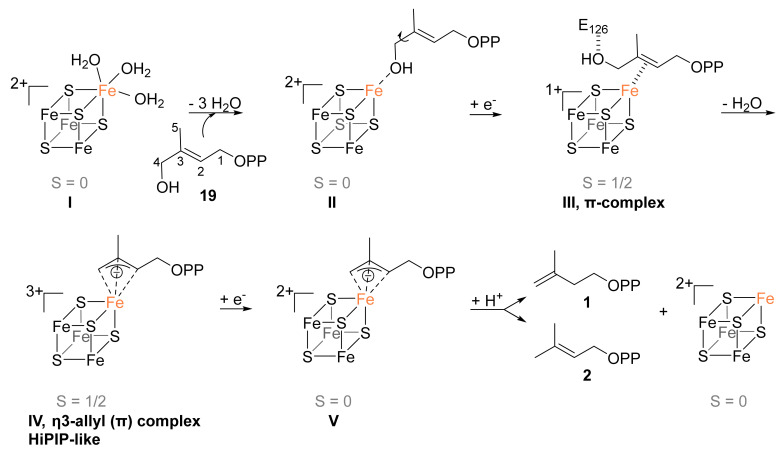
Proposition for the mechanism of the IspH catalyzed reductive dehydroxylation.

The proposed mechanistic course of the IspH enzyme is depicted in Scheme 2. Formally, the mechanism of IspH involves the elimination of water and the transfer of two electrons as well as two protons.

The active site of this reaction includes the [4Fe-4S] cluster that has already been described in detail in Section 3.3. Synthetic models for such 3:1 site-differentiated clusters were recently provided by the Suess group and it was shown that the unique fourth iron site could easily undergo ligand exchange [51] while the other ligands of the cluster were cysteine thiolates that are soft bases making stronger coordination bonds. It was proposed that the coordination geometry and the exchange at the unique iron site was controlled by steric effects provided by the ligands of the adjacent iron sites. Therefore, the opening of the coordination sphere of the apical iron required for its hexacoordination in IspH might be controlled by steric effects provided by the position of the cysteine ligands. Furthermore, broken-symmetry density functional theory (BS-DFT) studies performed on IspH indicated that the Fe-S bonds of the unique fourth iron site could be distorted which led to greater flexibility in ligand binding [52]. The three water molecules are good leaving groups contrary to the deprotonated cysteines exhibiting strong coordination bonds, suggesting a role of the apical iron of IspH in catalysis.

Docking studies of HMBPP using the *A. aeolicus* IspH structure in its [3Fe-4S]^+^ form in which the missing iron was computationally added revealed that the OH of HMBPP could bind to the fourth iron [36]. This observation was first experimentally confirmed using Mössbauer spectroscopy by adding the substrate HMBPP to ^57^Fe-LytB in solution [33]. A change in the isomer shift from δ_3_ = 0.89 mm s^−1^ to δ_3′_ = 0.64 mm s^−1^ of the component related to the unique fourth iron site of the cluster is observed in the presence of HMBPP, confirming the binding of the substrate to this iron site. The quadrupole splitting of this iron is also lowered from ΔE_Q,3_ = 1.97 mm s^−1^ to ΔE_Q,3′_ = 1.00 mm s^−1^ which indicates a change in the geometry of this iron ion. Upon substrate binding, the three water molecules of **I** are released and instead, the hydroxyl group of HMBPP is bound to the unique fourth iron site which is now tetrahedrally coordinated (intermediate **II**).

*E. coli* wild type or IspH mutants have been further crystallized with various ligands such as substrate, substrate analogs, products, or inhibitors (Figure 2, Table 3) [37,45,46,47,48,49,50]. In 2010, Groll, Bacher, and collaborators reported the first *E. coli* IspH structure with an intact [4Fe-4S] cluster as a complex with a substrate molecule (HMBPP; PDB ligand ID: H6P) [37]. (The ligand reported in the PDB file (3KE8) is not the substrate but an analog with the PDB ligand ID EIP, Figure 2, Table 3). The binding of HMBPP does not induce any significant backbone differences compared to the previously described structures. The diphosphate moiety inserts into a polar pocket composed of the side chains H41, 74, and 124, S225 and 269, T168, N227, and Q166 and is engaged in an extensive hydrogen bonding network. The substrate adopts a hairpin-like conformation to allow its C4 oxygen to form a coordination bond with the apical iron of the [4Fe-4S] cluster. The structures of the complex with each of the two reaction products (DMAPP and IPP) are also available [37]. Here again, the two molecules retain the same binding mode and their hairpin conformations but they are no longer bound to the apical iron.

The X-ray analysis of IspH in complex with its substrate confirmed that the hydroxyl moiety of HMBPP or its deprotonated form (the alkoxide) is bound to the unique fourth iron atom. With regards to the question of protonation, Blachly, Noodleman, and co-workers gave a plausible answer. By applying BS-DFT calculations, the protonated OH-form of HMBPP yields Mössbauer values comparable to the simulated values from experimental spectra [53]. Indeed, when HMBPP is bound to the apical iron as an alcohol, the HO-Fe distance is elongated with shortening of the distances of this iron and the S^2−^ giving to the apical iron more ferrous character as revealed by Mössbauer spectroscopy.

Together these results show that the first step in the mechanism of IspH is the binding of the OH of the substrate to the unique iron site accompanied by the release of three water molecules. It is not clear what triggers the departure of the three water molecules but conformational change of the protein upon binding of the HMBPP substrate might generate movement of the cysteine ligands leading to reduction of the space around the apical iron and therefore favoring tetrahedral coordination geometry. Similar steric effects were reported in models [51]. Furthermore, the hydrogen bonding network revealed in the IspH structure in complex with the substrate will also increase the stability of this complex compared to the substrate-free enzyme harboring loosely bound water molecules.

### 4.3. Second Step: Reduction of the [4Fe-4S]^2+^ Cluster and Rotation of the CH_2_OH Group

Systematic studies by Liu et al. with different organic redox mediators along with the reducing agent dithionite revealed that the enzyme’s activity was dependent on the redox potential of these redox mediators [34]. The enzyme was most efficient when using a system of dithionite and a mediator with a redox potential of −450 mV vs. NHE while no detectable activity was found for redox mediators bearing redox potentials above −250 mV vs. NHE or below −720 mV vs. NHE. By using this chemical system, the authors reported an activity of 31.6 µmol min ^−1^ mg ^−1^ which is much higher than the activity of about 800 nmol min ^−1^ mg ^−1^ that is now frequently described for *E. coli* IspH using the natural NADPH/flavodoxin reductase/flavodoxin as reduction system [32,33].

**Figure 3:**
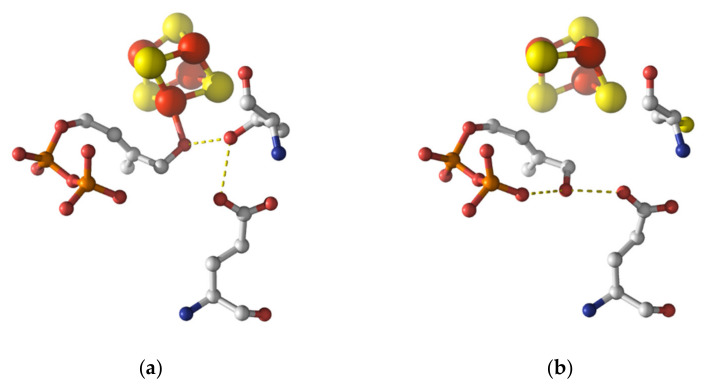
Crystallographic structures depicting the rotation of the hydroxymethyl group [45]. (**a**) The IspH WT was incubated with the substrate HMBPP. The full iron-sulfur cluster is present and the CH_2_OH group is coordinated to the apical iron, stabilized by T167. (**b**) The IspH T167C mutant was incubated with the substrate HMBPP. The CH_2_OH group has rotated about almost 180° and is now stabilized by the diphosphate group as well as the glutamate E126. The crystal structure only shows a [3Fe-4S] cluster.

After binding of the substrate to the apical iron atom, a rotation of the CH_2_OH group has been proposed upon reduction of the [4Fe-4S]^2+^ cluster to form intermediate **III**. The first who observed this rotation to be a part of the catalytic course were Oldfield et al. who conducted docking calculations of the substrate with reduced IspH [54]. After molecular mechanics optimization in silico, the hydroxyl group did not bind to the iron-sulfur cluster anymore but pointed towards the opposite direction as is depicted in Figure 3.

Intermediate **III** was then observed experimentally by Groll and collaborators who provided crystal structures of *E. coli* IspH enzyme with bound HMBPP [45]. The authors compared the structures before and after irradiation with X-rays as synchrotron irradiation is known to reduce some metalloenzymes [55,56]. Based on the measured electron density of the crystal, two HMBPP conformers were suggested: the early intermediate in which the OH was bound to the apical iron and a new conformer with the CH_2_OH group partially rotated away from the apical iron. The hydroxyl group was now stabilized intramolecularly by the pyrophosphate group as well as by the amino acid residue E126 of IspH, which in turn formed a hydrogen bond with T167. However, the interpretation was still ambiguous as the initial binding of the hydroxyl group to the iron-sulfur cluster could also be detected.

Independently, Dickschat and collaborators conducted feeding experiments with deuterated isotopologues of the precursor of the DOX pathway, 1-deoxy-D-xylulose [57]. These in vivo experiments were conducted on the bacteria *Streptomyces avermitilis* that are known to produce the isoprenoid pentalenene for which the biosynthetic route was deciphered. After conversion, the configuration of the IPP and DMAPP products was determined by tracing back the course of the deuterium atoms in pentalenene. Given that only one of the two possible deuterated IPP analogs was formed, a rotation of the CH_2_OH group seemed likely to be involved in the formation of the required intermediate.

More evidence for this rotation was given by HYSCORE experiments on the *E. coli* IspH E126Q mutant incubated with ^17^O-labeled HMBPP ([4-^17^O]HMBPP) [49]. In previous experiments, this mutant exhibited only 0.3% activity compared to the wild type [45]. It was proposed that E126 played a role in the proton transfer step, so mutation to glutamine might hinder the subsequent protonation needed for water elimination. After reduction of the [4Fe-4S]^2+^ cluster of IspH, an intermediate species was detected with a very low ^17^O hyperfine coupling constant (*A_iso_* = 1 MHz) compared to those of direct Fe-O bonds [49]. This led the authors to the conclusion that the substrate’s oxygen atom must have had a greater distance to the apical iron atom, most likely as a result of a rotation of the CH_2_OH group. The same authors had previously suggested that based on ^13^C-ENDOR experiments using the inactive E126A IspH mutant and [U-^13^C_6_]HMBPP a π- or π/σ-complex between the reduced cluster and the C2-C3 double bond of the substrate was formed. A similar complex had already been confirmed for a nitrogenase FeMo cofactor mutant in complex with allyl alcohol or ethylene [58,59]. The *g*-values of the IspH E126Q as well as the IspH E126A mutant were comparable with those of the Fe alkenyl complexes [49,54].

Similarly to the in vivo experiments, deuterated substrates were later prepared for more straightforward in vitro experiments with IspH [60]. HMBPP was deuterated on the C4-position to yield the two stereoisomers (*S*)-[4-^2^H_1_]HMBPP and (*R*)-[4-^2^H_1_]HMBPP, respectively. After incubation of each substrate with IspH in the presence of its naturally occurring reducing system, the configuration of the deuterium atoms on the products was determined by NMR spectroscopy. The *S*- and the *R*-configured substrates both yield [4-^2^H_1_]DMAPP without any stereocenters. However, the (*S*)-configured substrate only yields (*E*)-[4-^2^H_1_]IPP whereas the (*R*)-configured substrate only yields (*Z*)-[4-^2^H_1_]IPP. With regard to these results, Seemann and coworkers concluded that after reduction of the iron-sulfur cluster the CH_2_OH group rotated by almost 180° compared to the initial binding of the hydroxyl moiety to the apical iron atom as suggested from the previous structural data [45].

The driving force of this rotation may be explained by the fact that the apical ferrous ion becomes even softer upon reduction of the [4Fe-4S]^2+^ cluster [60]. According to the HSAB concept [61], this iron is more prone to form a complex with the soft alkene than with the harder hydroxyl group. The flexibility of the [4Fe-4S] cluster has been described in great detail by Holm et al. [62] and the reduced form of the cluster exhibits a greater core volume and distortions facilitating the ligand exchange. An η^2^-ring conformation of HMBPP was suggested by Blachly, Noodleman and coworkers [52]. The rotated CH_2_OH-group may be stabilized by forming intramolecular hydrogen bonds with the diphosphate moiety which was found to be the energetically most favorable configuration according to BS-DFT calculations when the [4Fe-4S] is reduced by one electron. Another possibility could be the formation of an η^2^-trans state where the CH_2_OH group is stabilized by the nearby E126. When the [4Fe-4S] is reduced by one electron both η^2^ configurations were showed to be lower in energy than the configuration having the hydroxyl of the substrate bound to the apical iron.

An alternative pathway where rotation of the CH_2_OH-group occurs before the reduction has also been proposed based on computed reduction potentials obtained from BS-DFT calculations [52].

### 4.4. Third Step: Protonation of the Hydroxyl Group and Elimination of Water

**Scheme 3:**
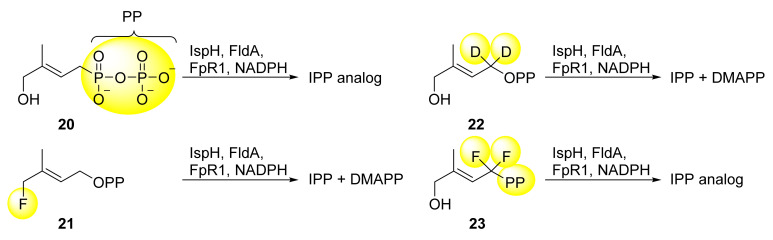
Substrate analogs of HMBPP used for the study of C-O bond cleavage [63,64]. Discrepancies from the natural substrate HMBPP are highlighted in yellow.

Mechanistic studies with substrate analogs were conducted by Liu et al. in order to get insights into the presumed C-O bond cleavage of the substrate’s hydroxyl group [63]. Replacing the diphosphate group at the C1 atom with a pyrophosphonate group (see Scheme 3, compound **20**) still leads to the IPP analog after incubation with IspH and the naturally occurring reducing system as confirmed by ^1^H NMR spectroscopy. Thus, C-O bond cleavage at the C1 site of HMBPP can be excluded despite the fact that diphosphate is a better leaving group than the hydroxyl group. When incubating the fluorine analog **21** with the system mentioned above both IPP and DMAPP are obtained. Hence, the mechanism is thought to be analogous to that for HMBPP. As the fluorine atom exhibits a high electronegativity, only a heterolytic cleavage of the C-F bond comes into consideration which is assumed for the C4-O bond as well. Furthermore, isotopic labeling of the C1 hydrogen atoms with deuterium (compound **22**) or substitution of hydrogen with fluorine (compound **23**) still allows some turnover of the substrate, and at least one of the two products (the IPP analog in case of compound **23**) are synthesized [64]. Therefore, it is unlikely that the C1 atom is deprotonated or otherwise directly involved in the mechanistic pathway.

As mentioned earlier, a possible proton source for the formation of water after hydroxyl elimination may be the glutamate residue E126. It lies in proximity to the hydroxyl group [45] and upon mutation to glutamine, aspartate, or alanine, the respective mutants E126Q, E126D, and E126A exhibit almost no activity [37,42,54]. The proton may be transferred to this glutamate residue by the adjacent histidine residue H124. It was shown that the respective mutants alanine (H124A) or phenylalanine (H124F) had a very low activity also [40,54]. Quantum mechanistics/molecular mechanistics (QM/MM) investigations support evidence that the dehydroxylation is triggered by a proton transfer from protonated E126 to the hydroxyl group of HMBPP mediated by a conserved water molecule [65].

### 4.5. Fourth Step: Formation of a HiPIP-like Cluster

The next intermediate (**IV**) was first observed by Duin et al. by *cw* EPR spectroscopy [40]. It was trapped by incubation of one electron-reduced IspH with its substrate without further electron source for complete turnover. The resulting paramagnetic species had *g*-values of 2.173, 2.013, and 1.997, comparable to those of ferredoxin:thioredoxin reductase that contains a [4Fe-4S]^3+^ cluster [66]. The authors proposed that the intermediate in the IspH catalyzed reaction was linked to [4Fe-4S]^3+^, but further interpretation of the data remained obscure.

Oldfield et al. clarified the nature of this intermediate. By freeze-quenching a reaction mixture containing IspH, dithionite as the only reducing agent, and the substrate, the reaction was slowed down and the trapped intermediate **IV** could be analyzed by EPR spectroscopy. Eventually, they measured the same intermediate as did Duin et al. [49]. The *g*-tensor was found to be *g* = [2.171, 2.010, 1.994] and was also similar to that found in other high-potential iron-sulfur proteins (HiPIP) [67]. The authors confirmed the presence of a [4Fe-4S]^3+^ cluster, which implicates that formally a two-electron transfer from the cluster to the substrate and the elimination of the hydroxyl group would occur.

**Figure 4:**
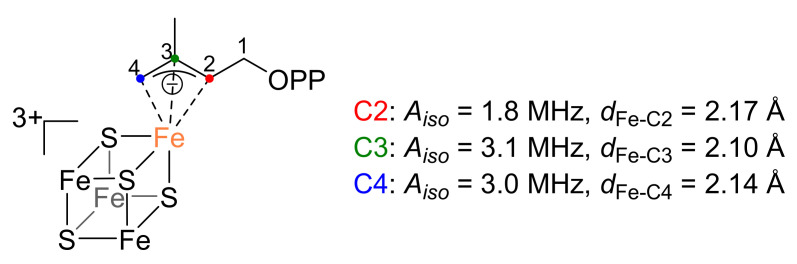
Schematic representation of the HiPIP-like η^3^-allyl (π/σ) complex. The hyperfine coupling constants *A_iso_* obtained after HYSCORE measurements of ^13^C-labelled HMBPP as well as the iron-carbon distances *d*_Fe-C_ estimated by DFT calculations are given for the C2, C3, and C4 atom [68].

Support for this [4Fe-4S]^3+^-intermediate was provided by the same group after HYSCORE experiments on one-electron reduced IspH incubated with HMBPP [68]. In these experiments, the electron source for the second electron transfer was again removed before incubation with the substrate, thus a possible reaction intermediate could be trapped. For the HYSCORE measurements, the oxygen of the substrate’s hydroxyl group had been isotopically labeled with ^17^O. As no ^17^O hyperfine coupling was detected, it was suggested that in the intermediate under discussion the hydroxyl group had already been eliminated. Furthermore, in another experiment one of the respective C2, C3, and C4 atoms of HMBPP had been labeled with ^13^C. In this way, possible hyperfine couplings to the unique fourth Fe species of the cluster could be measured. The ^13^C labeling led to hyperfine coupling constants of *A_iso_* = 1.8 MHz (C2), *A_iso_* = 3.1 MHz (C3) and *A_iso_* = 3.0 MHz (C4) (Figure 4). By DFT calculations, Fe-C distances of 2.17, 2.10, and 2.14 Å for the respective C2, C3, and C4 atoms were estimated in silico. As simulated *A_iso_* values were in the range of the experimental values, the proposed distances were taken as a good approximation and suggest an allyl anion that forms an η^3^-complex with the [4Fe-4S]^3+^ cluster. The Fe-C distances of 2.6–2.7 Å measured previously in the crystal structure of IspH in complex with the “converted substrate” reported by Gräwert et al. presumably represent this same intermediate [37]. The HiPIP-like cluster in complex with the delocalized π-system is considered as one of the rare examples of a bioorganometallic intermediate found in nature.

The formation of the η^3^-complex occurs after two-electron transfer from the cluster to the substrate and the elimination of the hydroxyl group. It is still unclear if the OH leaves the substrate as OH or as water after protonation by E126 and if the two-electron transfers are concomitant with the water formation.

### 4.6. Fifth Step: Protonation and Formation of the Two Products IPP and DMAPP

IspH catalyzes the reductive dehydroxylation of HMBPP into IPP and DMAPP with a ratio of about 4–6:1 [23]. This ratio is different from the equilibrium provided by isopentenyl diphosphate isomerase that favors the thermodynamically more stable DMAPP with a ratio of 3:7 (IPP:DMAPP) [24]. Obviously, protonation on the C2 atom of the allyl anion complex yields IPP while on the C4 atom, it yields DMAPP. The source of proton is still a matter of debate and several hypotheses for the proton mediator were done.

The proton source was identified early by Rohmer and co-workers without knowledge of the IspH-catalyzed mechanism [69,70] using the bacterium *Zymomonas mobilis* that due to its peculiar metabolic pathways (metabolization of glucose according to the Entner-Doudoroff pathway and no conversion of pyruvate into glyceraldehyde phosphate) is able to synthesize the two possible NAD(P)^2^H isotopomers from [1-^2^H]glucose. After feeding *Z. mobilis* with [1-^2^H]glucose, two pentacyclic triterpenes of the hopanoid series were analyzed by ^1^H- and ^13^C-NMR spectroscopy. These hopanoids exhibited deuterium atoms on definite positions of the carbon skeleton that could be traced back to the C2 and C4 positions of IPP and DMAPP. While the deuterium atom on the C4 position could be explained by an earlier reduction step in the MEP pathway catalyzed by DXR, the deuterium atom on the C2 position revealed the signature of a further reduction step in the formation of IPP and DMAPP that could not be explained at that time. The authors also observed that the deuterium was introduced in the pro-*S* position of IPP. Later on, the authors concluded that the deuterium was introduced by IspH and that NADPH was the proton donor for this catalytic step at least for this bacterium [28,71]. Feeding experiments of different methylerythritol isotopomers in *E. coli* [69] or of labelled deoxyxylulose in *E. coli* and in several plant cells [71] by Arigoni and co-workers and by the groups of Rohmer and Bach shed light on the introduction of a deuterium on C2 of IPP in the pro-*S* position [71,72,73,74].

**Scheme 4:**
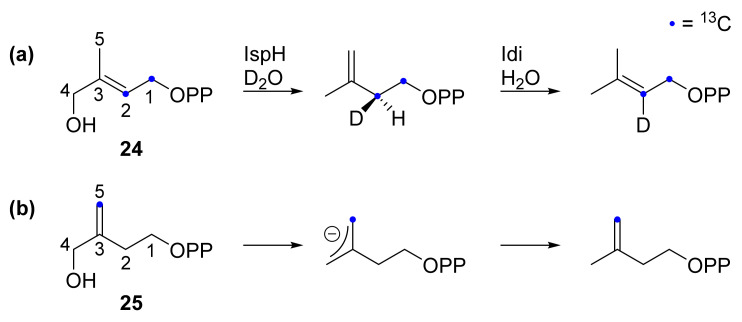
(**a**) Stereochemical course of deuterium and hydrogen after incubation with IspH in D_2_O and isopentenyl diphosphate isomerase in H_2_O [75]. (**b**) Substrate analog **25** used by Liu et al. and the product formed in the IspH catalyzed reaction [76].

The stereochemical course of the IPP formation was further investigated by Rohdich, Eisenreich et al. using enzymology on IspH [75]. The authors conducted ^13^C-NMR spectroscopy on isotopically labeled HMBPP (**24**) after incubation with IspH in D_2_O followed by incubation with isopentenyl diphosphate isomerase in H_2_O (Scheme 4a). The *E. coli* IPP isomerase is known to eliminate the pro-*R*-proton of IPP. The resulting DMAPP exhibits a deuterium atom on the C2 atom which thus corresponds to the pro-*S*-proton of IPP. Hence, it is proposed that protonation occurs only from the *si* face of the allyl anion. Based on the crystallographic structure, the diphosphate moiety of the substrate may serve as proton mediator on this site as it is in close vicinity to the C2 and C4 atoms [37,42].

In line with these assumptions, the product formation of the ^13^C isotopically labeled substrate analog **25** was investigated and it was found that only the IPP analog was formed [76] showing that the protonation was sterically restricted to the C4 atom. The authors explained this observation by the higher distance between the C5 of **25** and its diphosphate once **25** was in the active site of IspH that would prevent the diphosphate to protonate C5.

## 5. IspH Inhibitors

Since the first breakthrough into the IspH mechanism, it has been readily exploited for the generation of potent inhibitors to develop unprecedented and safe therapeutics to fight against multiresistant bacteria and the malaria parasite *Plasmodium falciparum.*

Most of the compounds which have been classified as IspH inhibitors interact with the apical iron of the [4Fe-4S] cluster through different mechanisms. The group of Hirsch demonstrated that the active site and the neighboring pockets of IspH are druggable and, moreover, molecules larger than the natural substrate might be able to access this spacious site [77]. All the inhibitors studied so far can be divided into four classes: substrate analogs, pyridine diphosphates, alkyne derivatives, and non-diphosphate compounds.

### 5.1. Analogs of the HMBPP Substrate as Inhibitors

**Figure 5:**
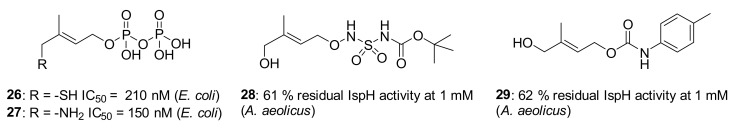
Substrate analogs as inhibitors of IspH.

The first step in the IspH mechanism is the binding of the hydroxyl group of the substrate to the apical iron (Fe^2+^) site of the [4Fe-4S]^2+^ cluster. Replacement of this OH by a softer ligand such as a thiol was envisioned. Furthermore, substrate analogs not able to undergo the OH elimination step such as amino analogs were considered. In this context, Poulter, Seemann, and collaborators conceived and tested two HMBPP analogs where a thiol or an amino group replaces the hydroxyl group of the substrate (Figure 5) [78,79]. A complete kinetic investigation under anaerobic conditions revealed that the thiol analog **26** and the amino analog **27** were extremely potent inhibitors of *E. coli* IspH displaying competitive mode of inhibition with K_i_ values in the nanomolar range (K_i_ = 24 nM for **26** and K_i_ = 54 nM for **27**). Furthermore, **27** presented a slow binding inhibition mode with the slow step being the formation of the enzyme-inhibitor complex. This might be explained by the fact that the amino group undergoes a deprotonation at physiological pH to bind to the apical iron, therefore, the authors assumed that the slow step was the deprotonation of the amine [78]. Previous Mössbauer spectroscopy studies showed a transition from octahedral to tetrahedral geometry for the apical iron when adding **26** or **27** to the enzyme, as it was already observed when HMBPP is in complex with IspH [79]. This finding validates that these inhibitors are bound via their amino and thiol group to the unique iron of the [4Fe-4S] cluster. The same binding mode was observed in the crystal structures of *E. coli* IspH in complex with these two potent inhibitors (AMBPP **27**; PDB ligand ID: 10E and TMBPP **26**; PDB ligand ID: 10G), confirming that **27** is bound to the apical iron of the [4Fe-4S]^2+^ cluster by its amino group and **26** is bound to this iron by its thiol group [47,48].

**Figure 6:**
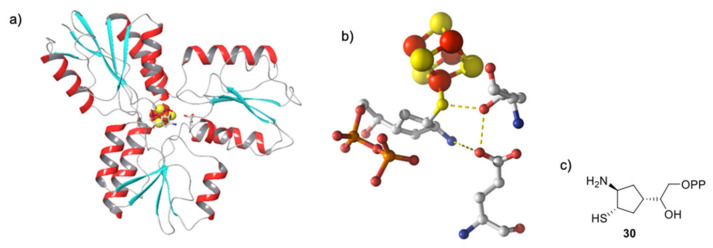
In silico docking result of the IspH inhibitor candidate **30** designed based on the structure of *E. coli* IspH:**26** (PDB ID: 3ZGN) and showing the best docking score. (**a**) Docking result of **30** binding to IspH; (**b**) active site; (**c**) chemical structure of **30**.

Nuclear resonance vibrational spectroscopy (NRVS) data were further recorded by Schünemann and collaborators for IspH bound to either of these inhibitors [38]. The measured NRVS data and those calculated based on the X-ray structure reported by Span et al. [48] showed reasonable agreement and the deviation was attributed to the presence of different IspH conformations in protein crystals and in solution. These differences were better explained once Borel and collaborators reported new structures of *E. coli* IspH in complex with these inhibitors in which the authors ascertained that the apical iron was present with an occupancy of 100% [47] as 35–45% of IspH molecules in the previously reported [48] *E. coli* IspH structure in complex with the amino analog **27** seemed to have an incomplete cluster. Furthermore, a detailed analysis of the *E. coli* IspH:**26** complex revealed that **26** bound in each monomer adopted two different conformations [47]: a classical one, superimposed on HMBPP already described, and one in which the C4 atom of **26** was pulled away from the diphosphate group and E126. The fact that IspH can accommodate more than one substrate conformer was proposed to be related to the selectivity of the protonation and consequently to the genesis of the two products. The presence of two conformers was further exploited to generate in silico putative IspH inhibitors with enhanced affinity by mimicking the space occupied by both conformers by a cyclopentyl group linked to a thiol and a diphosphate moiety [47]. Interestingly, the best virtual inhibitor candidate (**30**) that arose from these in silico evolution experiments is displayed in Figure 6 and revealed binding of the thiol moiety to the apical iron of the [4Fe-4S]^2+^ cluster and formation of a salt bridge between an amino group and E126. However, no data of the potency of inhibition of **30** on IspH was reported.

Two more substrate analogs have been designed by the group of Van Calenbergh and the group of Jomaa by replacing the diphosphate of HMBPP with a *N*-acyl-*N*′-oxy sulfamate function leading to **28** and a carbamate moiety leading to **29** (Figure 5). These compounds, tested at 1 mM concentration, showed a very low inhibition of IspH from *A. aeolicus* [80]. The poor inhibition potency of both molecules might be related to the fact that they lack the diphosphate group which is involved in important interactions with different histidine residues, such as His42 and His124 that are highly conserved in IspH of several species [36].

### 5.2. Pyridine Diphosphate as Inhibitors

**Figure 7:**
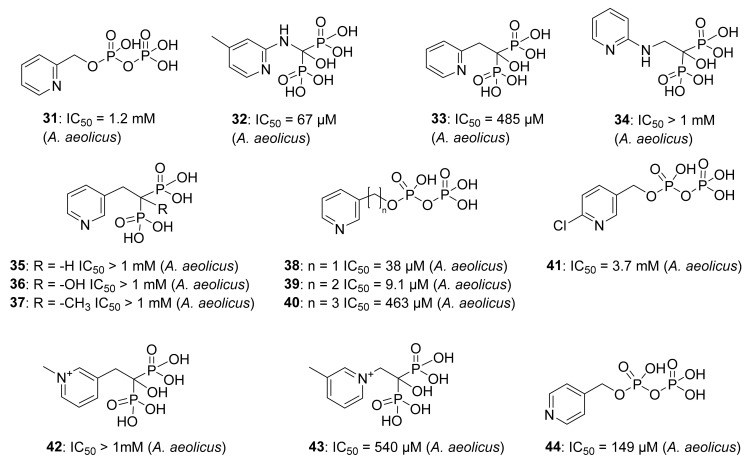
Compounds **31**–**44**, tested as inhibitors of IspH from *A. aeolicus*.

**Figure 8:**
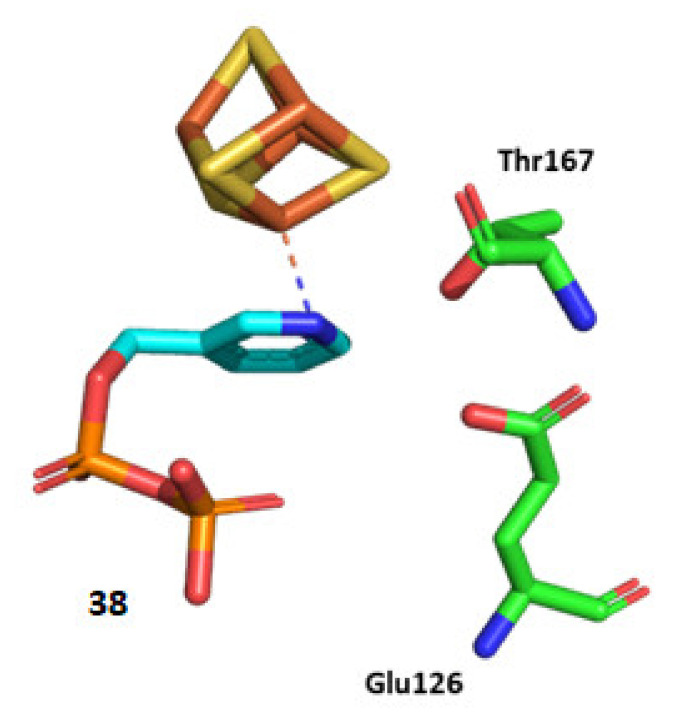
Structural representation of *Ec*-IspH in complex with compound **38** (PDB code 4MUX) [50]. The amino acids are shown as sticks, as well as the [4Fe-4S] cluster and the ligands.

Wang et al. studied a set of pyridine derivatives substituted in ortho, meta, and para (**31**–**44**) (Figure 7), as potential inhibitors of IspH from *A. aeolicus* [81]. In some compounds, the diphosphate has been replaced by the bioisostere bisphosphonate, which is more stable to phosphatase hydrolysis in vivo [82]. ^14^N and ^15^N HYSCORE investigations by Oldfield and co-workers demonstrate that inhibitor **38** binds to the apical iron probably via a η^1^ bonding between the nitrogen and the [4Fe-4S] cluster, whereas no ^14^N hyperfine interactions were observed with ortho and para pyridinyl analogs [83]. Furthermore, crystallographic studies reported by Span, Groll, Oldfield, and collaborators confirmed that the pyridine is close to the apical iron (2.3–2.4 Å), and the authors observed a continuous electron density between the nitrogen of the pyridine derivative and the apical iron. According to these observations and quantum chemical calculations, Span et al. provided evidence that the binding occurs through a η^2^ coordination [50]. Moreover, the authors explored the orientation that the pyridine ring adopts in relation to the [4Fe-4S] cluster. X-ray crystallographic studies on *E. coli* IspH demonstrated that the nitrogen of the pyridine points in a direction likely represented in Figure 8. This orientation is presumably the result of the interaction of **38** with two pockets of the catalytic site, a lipophilic one characterized by four Val15, Val43, Val73, Val99 residues, and a hydrophilic site composed of Glu126, Thr167, Thr168, Thr200, and Asn227 residues [50].

Considering the insights acquired about the pyridine binding mode, it seems clearer why **38** and **39** showed the best inhibitory potency in this class. For example, **41** is not an inhibitor probably due to electron-withdrawing effect of the chlorine in *ortho*-position, which reduces the capacity of the nitrogen as electron donor Lewis-base. For similar reasons, the cation pyridinium analogs **42** and **43** did not show any biological activity.

### 5.3. Alkyne Derivatives as Inhibitors

**Figure 9:**
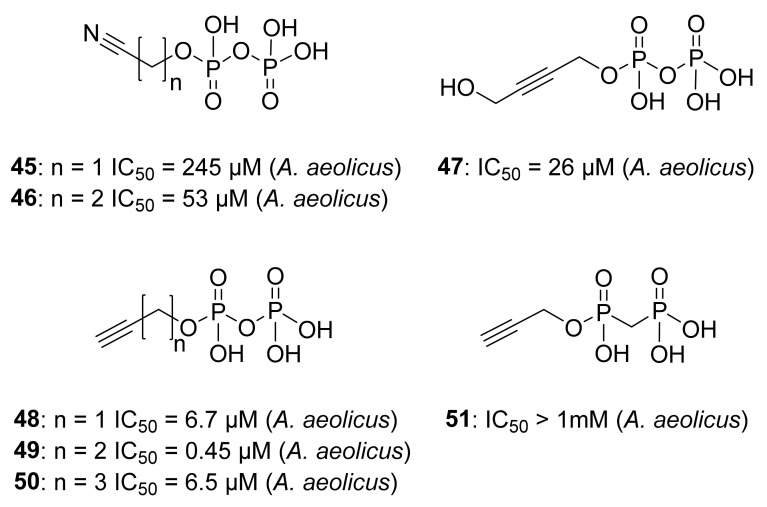
Compounds **45**–**51**, tested as inhibitors of IspH from *A. aeolicus*.

Wang et al. designed a series of alkyne diphosphates **45**–**50** and a phosphonate analog **51** (Figure 9), which were first screened by EPR spectroscopy. Furthermore, EPR investigations and specifically ^13^C-ENDOR results of reduced *A. aeolicus* IspH with [^13^C_3_]-**48** revealed strong ^13^C hyperfine coupling interactions (6 MHz) [54], allowing to conclude that the acetylenic diphosphates bind through the alkyne fragment to the [4Fe-4S]^+^ cluster by π or π-σ interactions. The *A. aeolicus* IspH inhibition assays demonstrated that **49** is the best inhibitor of this category (IC_50_ = 0.45 µM). The substitution of the diphosphate with a phosphonate for **51** led to a dramatic drop in inhibition potency, which might be due to the fact that the diphosphate fits better in the conserved cationic pocket seen in previous crystallographic investigations [37]. Moreover, Span et al. examined the compounds **48**–**50** bound to the oxidized IspH from *E. coli* by Mössbauer spectroscopy [46]. Previous investigations [33] helped to easily interpret the results obtained by the authors, then stating that the apical iron undergoes a conversion from octahedral to tetrahedral geometry with a 3S1O coordination, likewise what happens with the natural substrate HMBPP. Co-crystallographic structures of **48** with oxidized IspH were in accordance with the unexpected Mössbauer spectroscopy results and demonstrated that the binding between the alkyne residue and the fourth iron is not direct, indeed a water molecule is in between [46]. This result was further validated using NRVS measurements of the IspH-**48** complex [84]. NRVS studies of the free-substrate IspH had previously shown that the apical iron was bound to three sulfide ions of the cluster and to three molecules of water [38]. This finding was analog for the NRVS results of IspH in its [4Fe-4S]^2+^ state in presence of **48**, where the fourth iron was in a tetrahedral geometry not linked to the acetylene residue but to a single molecule of water.

On the other hand, due to the impossibility of studying the oxidized IspH by EPR, the inhibitor **48** was examined by EPR in complex with the reduced IspH. The results obtained by O’Dowd et al. [84] revealed plausible π-interactions between the inhibitor **48** and the reduced [4Fe-4S] cluster.

**Scheme 5:**
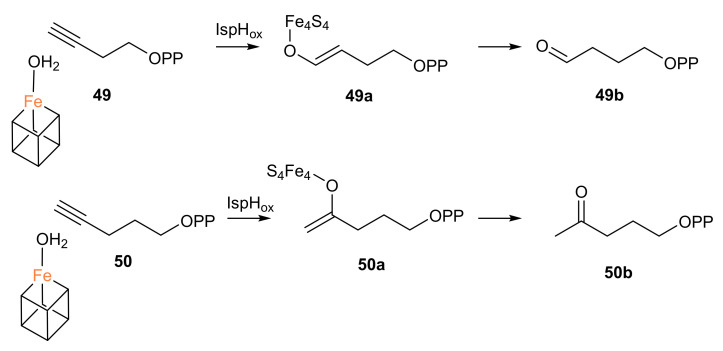
Reactions of the inhibitors **49** and **50** catalyzed by the enzyme IspH in the oxidized state. The ligand bound to the apical iron could also be an OH.

Surprisingly, X-ray structures of the best inhibitor **49** showed that IspH converts this compound to an aldehyde (Scheme 5), revealing two ligand conformations. One is an η^1^-O-enolate in complex with the apical iron, and the other one is a cyclic conformation of the aldehyde which binds to the Glu126 residue. Moreover, **50** was shown to react with the oxidized IspH, yielding in this case a ketone (Scheme 5). These unexpected products were also confirmed by ^1^H-NMR spectroscopy and electrospray ionization mass spectrometry (ESI-MS) [46].

### 5.4. Non-Diphosphate Inhibitors

**Figure 10:**
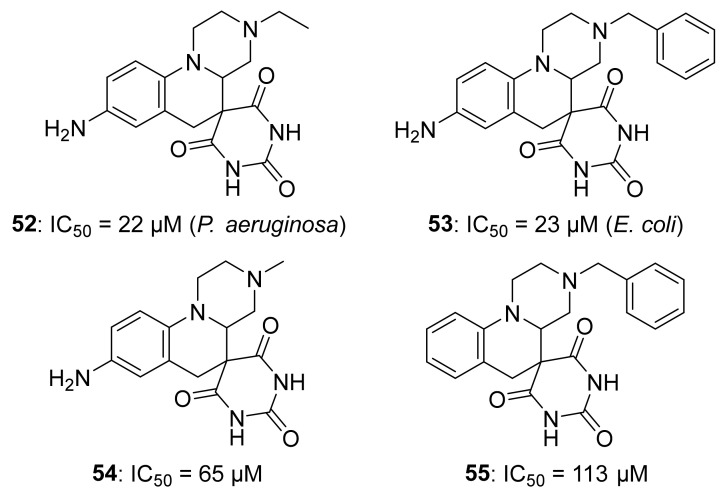
Compounds **52**– **55** as inhibitors of IspH. The origin of IspH for inhibitions assays of **54** and **55** was not mentioned by the authors.

O’Dowd et al. used an in silico approach to screen a series of compounds from ZINC and NCI libraries and after having tested some hits for IspH inhibition, they identified **52** and **53** as two new IspH inhibitors (Figure 10) [84]. In both compounds, it is possible to see a common barbituric acid sub-structure which makes the hits more drug-like, similarly to antibacterial agents developed by Pharmacia Corporation [85]. The compound **52** resulted to be a good inhibitor of IspH from *P. aeruginosa*, with an IC_50_ = 22 µM. On the other hand, the analog **53** inhibits IspH from *E. coli*, showing as result an IC_50_ = 23 µM [84]. After docking experiments of **53** into the *A. aeolicus* IspH structure (PDB ID: 3DNF), the authors proposed a plausible binding mode where the barbiturate might chelate the apical iron of the [4Fe-4S]^2+^ cluster, similarly to how barbiturate enolates do with Zn^2+^ in other metalloenzymes [86,87]. However, EPR studies for **53** in complex with the reduced IspH from *P. aeruginosa* did show a very low signal intensity, probably due to a change of the redox potential upon ligand binding or to a different relaxation mode [84]. These compounds could be developed as potential drugs for several reasons: they solved the problem related to the lability of the diphosphate moiety present in other inhibitors in vivo [82]; they respect the Lipinski’s rule [88] and they are not PAINS compounds [89]. Although they are promising drug-like compounds, further studies to validate the binding mode should be realized.

Potent IspH inhibitors have already been discovered with **26** and **27** showing the best inhibition properties. However, it should be noticed that no inhibitors having an activity on bacteria or apicomplexan parasites have been reported to date.

## 6. Conclusions

The oxygen sensitivity of IspH explains why the MEP pathway was overlooked for so many decades and not evidenced in the late 1950s when the mevalonate pathway was discovered by Bloch, Lynen, Cornforth. At that time, the researchers performed their enzymology experiments on the bench and did not have the equipment to work under anaerobic conditions nor the knowledge and spectroscopies needed for the characterization of metalloenzymes. Thanks to the advent of EPR, Mössbauer spectroscopy, and bioinorganic chemistry, it is now clear that IspH contains a peculiar [4Fe-4S]^2+^ cluster that is the active species and enables the electron transfer towards the substrate HMBPP in concert with a biological reduction system. Crystal structures, (labelled) substrate analogs synthesis and spectroscopic techniques permitted insights into the catalytic mechanism of IspH and led to the discovery of the first examples of bioorganometallic intermediates involving [4Fe-4S] centers. However, the complete mechanism of the IspH-catalyzed reaction is not fully understood. While it is now established that the first step is the binding of the substrate to the [4Fe-4S]^2+^ cluster, that a rotation of the OH of the substrate occurs upon reduction of the [4Fe-4S]^2+^ cluster and that the S = 1/2 η^3^-allyl-[4Fe-4S]^3+^ intermediate is formed, it is still unclear how the OH or water is removed and how the electron transfer occurs. As most investigations relied on paramagnetic species, no evidence of transient diamagnetic intermediates could be trapped. X-ray crystallography would be an elegant method to trap such intermediates. However, it suffers from the high lability of the apical iron and the obtained structures do not necessarily represent the intermediates present in solution. Obviously, Mössbauer spectroscopy will be a key player in the identification of such intermediates and more sensitive methods such as NRVS will be needed. Further investigations will also rely on the synthesis of new biomimetic models or other innovative molecular tools. The proton source involved in the formation of the two final products is still under discussion. Another question is the ratio of the products formed. What triggers the formation of about six times more of the less thermodynamically favored IPP compared to DMAPP? This raises also the question about the reduction systems. Why do some species use flavodoxin and others ferredoxin to transfer the electrons to the [4Fe-4S]^2+^ center of IspH? How are these electrons transferred in vivo? One puzzling question is the maintenance of an oxygen sensitive [4Fe-4S]^2+^ cluster in microorganisms living under aerobic conditions or in plant chloroplasts that produce O_2_ in phototropic conditions. Is the cluster continuously repaired? Are protecting enzymes involved? ErpA and NfuA proteins might be such candidates as they were shown to interact with IspH in *E. coli*, and NfuA was proposed to assist ErpA in Fe-S cluster delivery under oxidative stress [90].

Although some questions are still unanswered, most insights acquired about the mechanism of IspH were already exploited to discover promising inhibitors. However, none of these potent inhibitors were reported to be active on cells. Therefore, one of the big remaining challenges will also be the discovery of such molecules as they would represent good starting points to bring to the market innovative drugs with unprecedented modes of action that are urgently needed to fight against multidrug-resistant infectious diseases.

## Figures and Tables

**Table 1:** Occurrence of IspH in the bacteria classified as priority by the WHO.

Priority	Bacteria	IspH
critical	*Acinetobacter baumannii,* carbapenem-resistant	+
	*Pseudomonas aeruginosa*, carbapenem-resistant	+
	*Enterobacteriaceae*, carbapenem-resistant, ESBL-producing	+
high	*Enterococcus faecium*, vancomycin-resistant	− ^a^
	*Staphylococcus aureus*, methicillin-resistant, vancomycin-intermediate and resistant	−
	*Helicobacter pylori*, clarithromycin-resistant	+
	*Campylobacter* spp., fluoroquinolone-resistant	+
	*Salmonellae*, fluoroquinolone-resistant	+
	*Neisseria gonorrhoeae*, cephalosporin-resistant, fluoroquinolone-resistant	+
medium	*Streptococcus pneumoniae*, penicillin-non-susceptible	−
	*Haemophilus influenzae*, ampicillin-resistant	+
	*Shigella* spp., fluoroquinolone-resistant	+

^a^ partial IspH protein with incomplete catalytic site; rely on MVA pathway [14].

**Table 2:** Results of Mössbauer and NRVS spectroscopy on the [4Fe-4S]^2+^ cluster [33,38]. The Mössbauer spectra were recorded at T = 77 K and B = 0 T. The ΔE_Q_ and δ values of the HMBPP-bound IspH are found in parentheses.

	ΔE_Q_ [mm s^−1^]	δ [mm s^−1^]	Oxidation State and Coordination
Component 1 (50%)	1.21 (1.33)	0.42 (0.42)	Tetrahedrally coordinated Fe^2.5+^, mixed valence iron pairs, 3 S^2−^ and 1 Cys ligand
Component 2 (25%)	0.89 (0.92)	0.37 (0.38)	Tetrahedrally coordinated high-spin Fe^3+^, 3 S^2−^ and 1 Cys ligand
Component 3 (25%)	1.97 (1.00)	0.89 (0.64)	Hexa-coordinated high-spin Fe^2+^, 3 S^2−^ and 3 H_2_O (tetrahedrally coordinated, 3 S^2−^ and OH_HMBPP_)

**Table 3:** Crystallization conditions and structural data of the reported IspH.

PDB ID	Release Date	Source Organism	Crystallization Method (Vapor difusion)	Crystal Growth Procedure	pH	Space Group	Res. (Å)	Cluster	Ligand ID	Ref
3DNF	30 December 2008	*A. aeolicus*	Hanging drop	0.1 M Tris-Hcl, 10% PEG 8000	8.0	P 1 21 1	1.65	*Fe3 S4*	-	[36]
4N7B	20 November 2013	*P. falciparum*	Sitting drop	0.1 M Na citrate, 0.1 M (NH_4_)_2_SO_4_, 30% PEG 4000	5.6	P 31 2 1	2.20	*Fe3 S4*	SO4	[43]
3F7T	7 July 2009	*E. coli*	Hanging drop	1.6 M Potassium Phosphate	8.0	P 32 2 1	1.80	*Fe3 S4*	POP	[42]
3KEF	12 January 2010	*E. coli*	Sitting drop	0.1 M Bis-Tris, 0.2 M Li_2_SO_4_, 25% PEG 3350	6.5	P 21 21 21	1.70	*Fe3 S4*	DMA	[37]
3KEL							1.80	*Fe3 S4*	POP	
3KE8							1.70	**Fe4 S4**	EIP	
3KE9							1.90	**Fe4 S4**	IPE	
3KEM					7.0	C 1 2 1	2.00	*Fe3 S4*	IPE	
3T0G	30 November 2011	*E. coli*	Hanging drop	0.1 M Bis-Tris, 0.2 M Li_2_SO_4_, 25% PEG3350	6.5	P 21 21 21	2.10	*Fe3 S4*	H6P	[45]
3T0F							1.90	*Fe3 S4*	H6P	
3SZU							1.40	*Fe3 S4*	H6P	
3SZL							1.60	**Fe4 S4**	H6P	
3SZO							1.60	**Fe4 S4**	H6P	
3URK	5 September 2012	*E. coli*	Sitting drop	0.1 M Bis-Tris, 0.2 M (NH_4_)_2_SO_4_, 25% PEG3350	6.5	P 21 21 21	1.50	**Fe4 S4**	0CG	[46]
3UTC							1.90	**Fe4 S4**	0JX	
3UTD							1.70	*Fe3 S4*	OCJ	
3UV3							1.60	**Fe4 S4**	0CM	
3UV6							1.70	**Fe4 S4**	0CH	
3UV7							1.60	**Fe4 S4**	0CN	
3UWM							1.80	**Fe4 S4**	0K2/0JX	
3ZGL	9 January 2013	*E. coli*	Hanging drop	0.1M Bis-Tris, 0.2 M Li_2_SO_4_, 24% PEG 3350	6.5	C 1 2 1	1.68	**Fe4 S4**	10E	[47]
3ZGN							1.95	**Fe4 S4**	10G	
4H4C	23 January 2013	*E. coli*	Sitting drop	0.1 M Bis-Tris, 0.2 M (NH_4_)_2_SO_4_, 25% PEG3350	6.5	P 21 21 21	1.80	**Fe4 S4**	10D	[48]
4H4D							1.35	**Fe4 S4**	10E	
4H4E							1.70	**Fe4 S4**	10G	
4EB3	6 February 2013	*E. coli*	Sitting drop	0.1 M Bis-Tris, 0.2 M (NH_4_)_2_SO_4_, 25% PEG3350	6.5	P 21 21 21	1.90	**Fe4 S4**	0O3	[49]
4MUY	11 June 2014	*E. coli*	Sitting drop	0.1 M Bis-Tris, 0.2 M (NH_4_)_2_SO_4_, 25% PEG3350	6.5	P 21 21 21	1.80	*Fe3 S4*	2E5	[50]
4MV0							1.90	*Fe3 S4*	2E6	
4MV5	18 June 2014						1.90	*Fe3 S4*	2E7	
4MUX							1.70	**Fe4 S4**	2E4	
